# TIM-3 drives temporal differences in restimulation-induced cell death sensitivity in effector CD8^+^ T cells in conjunction with CEACAM1

**DOI:** 10.1038/s41419-021-03689-6

**Published:** 2021-04-14

**Authors:** Camille M. Lake, Kelsey Voss, Bradly M. Bauman, Katherine Pohida, Timothy Jiang, Gabriela Dveksler, Andrew L. Snow

**Affiliations:** 1 Department of Pharmacology & Molecular Therapeutics, Uniformed Services University of the Health Sciences, Bethesda, MD 20817 USA; 2 Henry M. Jackson Foundation, 6720A Rockledge Drive, Bethesda, MD 20817 USA; 3 Department of Pathology, Uniformed Services University of the Health Sciences, Bethesda, MD 20817 USA

**Keywords:** Cell death and immune response, Immune cell death, Cytotoxic T cells

## Abstract

Immune homeostasis depends upon effective clearance of pathogens while simultaneously preventing autoimmunity and immunopathology in the host. Restimulation-induced cell death (RICD) is one such mechanism where by activated T cells receive subsequent antigenic stimulation, reach a critical signal threshold through the T cell receptor (TCR), and commit to apoptosis. Many details of this process remain unclear, including the role of co-stimulatory and co-inhibitory proteins that influence the TCR signaling cascade. Here we characterize the role of T cell immunoglobulin and mucin domain containing 3 (TIM-3) in RICD regulation. TIM-3 protected newly activated CD8^+^ effector T cells from premature RICD during clonal expansion. Surprisingly, however, we found that TIM-3 potentiated RICD in late-stage effector T cells. The presence of TIM-3 increased proximal TCR signaling and proapoptotic protein expression in late-stage effector T cells, with no consistent signaling effects noted in newly activated cells with or without TIM-3. To better explain these differences in TIM-3 function as T cells aged, we characterized the temporal pattern of TIM-3 expression in effector T cells. We found that TIM-3 was expressed on the surface of newly activated effector T cells, but remained largely intracellular in late-stage effector cells. Consistent with this, TIM-3 required a ligand to prevent early RICD, whereas ligand manipulation had no effects at later stages. Of the known TIM-3 ligands, carcinoembryonic antigen‐related cell adhesion molecule (CEACAM1) showed the greatest difference in surface expression over time and also protected newly activated cells from premature RICD, with no measurable effects in late-stage effectors. Indeed, CEACAM1 enabled TIM-3 surface expression on T cells, implying a co-dependency for these proteins in protecting expanding T cells from premature RICD. Our findings suggest that co-signaling proteins like TIM-3 and CEACAM1 can alter RICD sensitivity at different stages of the effector T cell response, with important implications for checkpoint blockade therapy.

## Introduction

Restimulation-induced cell death (RICD) is a homeostatic process through which a proportion of T cells restimulated through their T cell receptor (TCR) succumb to apoptosis, enforcing peripheral tolerance and establishing an upper limit boundary of T cell expansion^1,2^. This process is imperative in the maintenance of healthy immune responses, as patients with X-linked lymphoproliferative disease due to SLAM-associated protein (SAP) deficiency (XLP-1) can perish from Epstein–Barr virus-induced fulminant infectious mononucleosis. Death is not due to viral pathogenesis, but rather attributed to unbridled CD8^+^ effector T cell expansion caused by a profound RICD defect, resulting in severe immunopathology^3^. RICD is thus critical in maintaining balance to ensure a healthy immune response against pathogens and prevent autoimmunity.

A number of mechanisms have been described which sensitize T cells to RICD. We and other groups have shown that the cell cycle phase^4^, IL-2 signaling^5^, metabolic state^6,7^, SAP and natural killer (NK), T, B cell antigen (NTB-A) expression^8^, diacylglycerol kinase alpha (DGKα) activity^9^, and transient expression of transcription factors such as forkhead box protein 3 (FOXP3)^10^ can all influence when and how conventional T cells are susceptible to RICD. One key driver of differential RICD sensitivity in late-stage effector T cells is relative TCR signal strength^11^; only T cells that reach a critical TCR signal threshold via strong, repeated restimulation will succumb to RICD. This insight suggests that proteins which directly regulate TCR signal strength should have a measure of influence over RICD sensitivity in effector T cells, though this has not been thoroughly investigated. Furthermore, the dependence on TCR signal strength is unclear in newly activated cells, in which the mechanisms driving RICD resistance remain largely unknown.

Coinhibitory proteins modulate T cell signaling in several normal and disease contexts, including the promotion of “exhaustion” in response to chronic infection or cancer^12,13^. Though they have a multitude of associated mechanisms, most coinhibitory receptors are thought to blunt T cell activation and function by directly or indirectly influencing TCR proximal signaling^14,15^. T cell immunoglobulin and mucin-domain containing-3 (TIM-3) is one such coinhibitory protein originally discovered as a marker of T helper 1 (Th1) CD4^+^ T cells, and now commonly associated with severely exhausted effector CD8^+^ T cells^16–22^. TIM-3 has been implicated in driving T cell apoptosis within the tumor microenvironment through the binding of its ligand, Galectin-9 (Gal-9)^23–25^. In some instances, antibody-mediated blockade of TIM-3 has been shown to reverse the exhausted state of these terminally differentiated cells as well as NK cells, demonstrating its potential as a target of checkpoint inhibition for cancer immunotherapy^26–28^. However, other reports demonstrate that only the co-blockade of TIM-3 with other similar checkpoint inhibitors brings about the full reversal of exhaustion in a clinical setting^28,29^. Though the full function of TIM-3 seems to vary depending on the context in which this protein is expressed, its role in fostering severe T cell exhaustion suggests potential mechanisms by which TIM-3 influences other natural immune homeostatic processes.

The purpose of this study was to ascertain whether TIM-3 could protect effector CD8^+^ T cells from RICD, which we hypothesized would be driven through a mechanism of TCR signal suppression. Strikingly, we found that TIM-3 influences RICD sensitivity differently at distinct phases of the T cell response: during early clonal expansion, TIM-3 works with the coinhibitory protein carcinoembryonic antigen‐related cell adhesion molecule (CEACAM1) to protect cells from premature RICD. In contrast, TIM-3 works independently of CEACAM1 to promote RICD in late-stage, terminally differentiated effectors entering the contraction phase, boosting TCR signaling to increase pro-apoptotic protein expression. This work demonstrates that “coinhibitory” proteins like TIM-3 and CEACAM1 participate in the temporal “tuning” of RICD sensitivity to influence T cell population dynamics.

## Results

### TIM-3 protects newly activated T cells from RICD, but exacerbates RICD in late-stage effectors

**Fig. 1:**
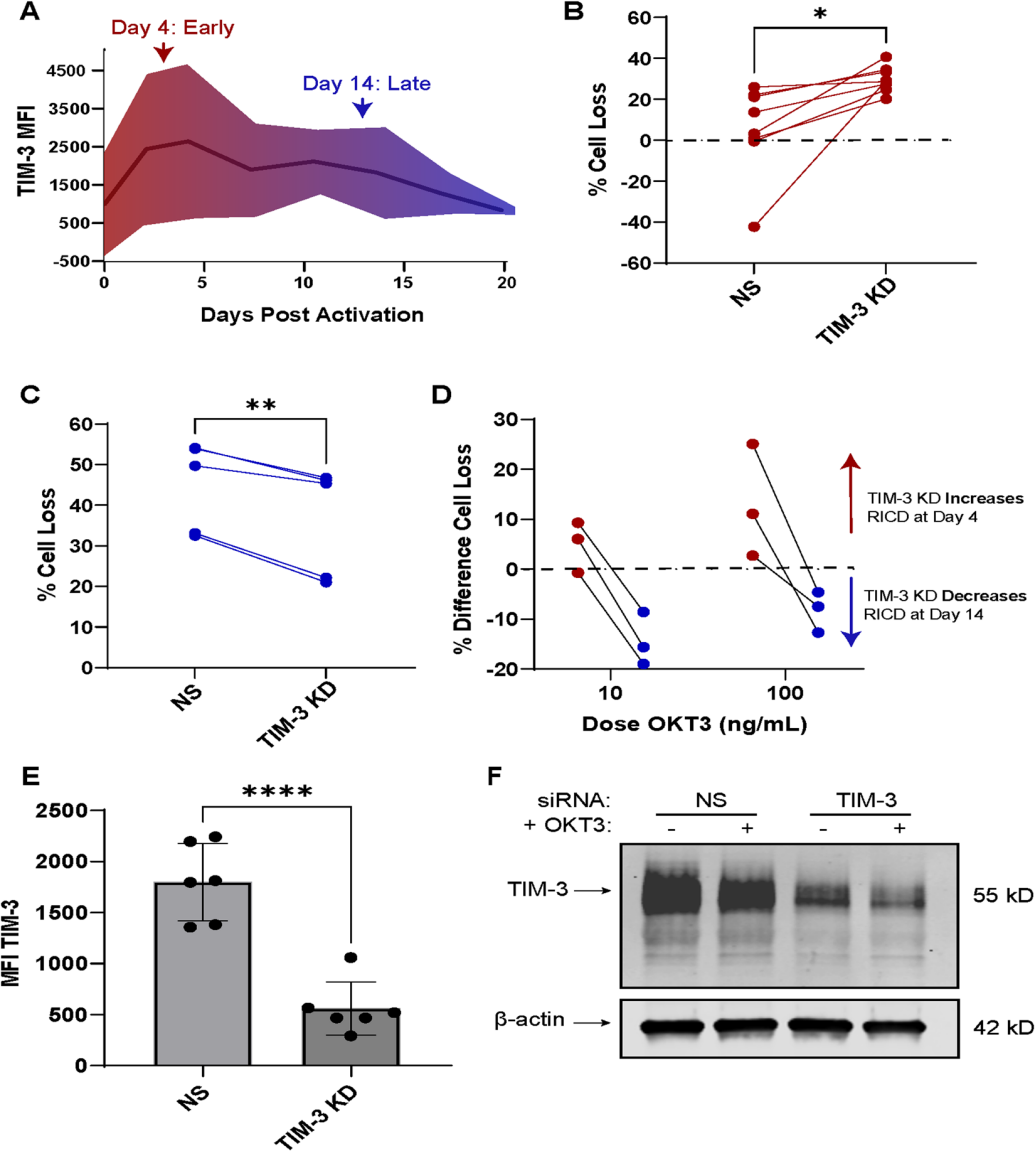
TIM-3 protects newly activated cells from RICD, but exacerbates RICD in late-stage effectors. **A** Isolated human CD8^+^ effector T cells were activated and cultured with exogenous IL-2 for up to 20 days post-activation. Representative image of 3 donors’ TIM-3 surface expression as assessed by flow cytometry; mean fluorescence intensity (MFI) is represented by the black line, outer edges represent standard deviation. **B** Purified CD8^+^ T cells were electroporated on day 0 with either TIM-3-specific siRNA (TIM-3 KD) or nonspecific siRNA (NS) and tested on day 4 for RICD sensitivity by restimulating with 100 ng/mL OKT3 antibody overnight. Cell loss was assessed by propidium iodide staining and flow cytometry. Connecting lines represent individual donors. **C** Cells were assessed as in (**B**) for death sensitivity at day 14 post-activation. **D** Differences in % cell loss, calculated per donor by subtracting NS % cell loss from TIM-3 KD % cell loss, were plotted for each donor at various concentrations of restimulating antibody OKT3. **E** Surface TIM-3 knockdown was assessed by flow cytometry; TIM-3 MFI of six representative donors are shown. **F** Cell lysates were collected following restimulation with OKT3 for 4 h and assessed for TIM-3 KD efficiency by immunoblotting; β-actin served as a loading control. Data shown are representative of several donors. Asterisks denote statistical significance using the following tests: **B** paired *t* test, *p* = 0.0156; **C** paired *t* test, *p* = 0.031; **E** paired *t* test, *p* < 0.0001.

TIM-3 has long been considered a marker of terminally differentiated, exhausted effector T cells^12,13,30^. However, some reports have shown that TIM-3 is expressed immediately post-activation of resting T cells, and can even enhance activation during both acute and chronic viral infections^31^. To first investigate at which time primary human CD8^+^ T cells express TIM-3, we assessed TIM-3 surface expression over a time course of 20 days post-activation. TIM-3 was expressed immediately post-activation of primary CD8^+^ T cells; variable expression across multiple donors waned over time in culture (Fig. 1A). Because TIM-3 was expressed at both early (red: days 0–6 post-activation) and late (blue: days 10–14 post-activation) time points in primary CD8^+^ effector T cells, we investigated the effect of TIM-3 siRNA-mediated silencing on RICD at both stages when cells are clonally expanding (day 4) or nearing contraction (day 14). Remarkably, for each donor tested, RICD increased following TIM-3 knockdown in newly activated effectors, and decreased following TIM-3 knockdown in late-stage effectors (Fig. 1B, C, respectively). This trend was apparent across individual donors at both early and late stages using various doses of restimulating antibody OKT3 (Fig. 1D), with a reliable TIM-3 knockdown efficiency of >50% (Fig. 1E, F). These data suggest that TIM-3 protects newly activated cells from premature RICD during clonal expansion, but helps facilitate RICD-mediated contraction of effector T cells after 14 days in culture.

### TIM-3 increases TCR signaling and expression of downstream apoptotic proteins in late-stage effectors

**Fig. 2:**
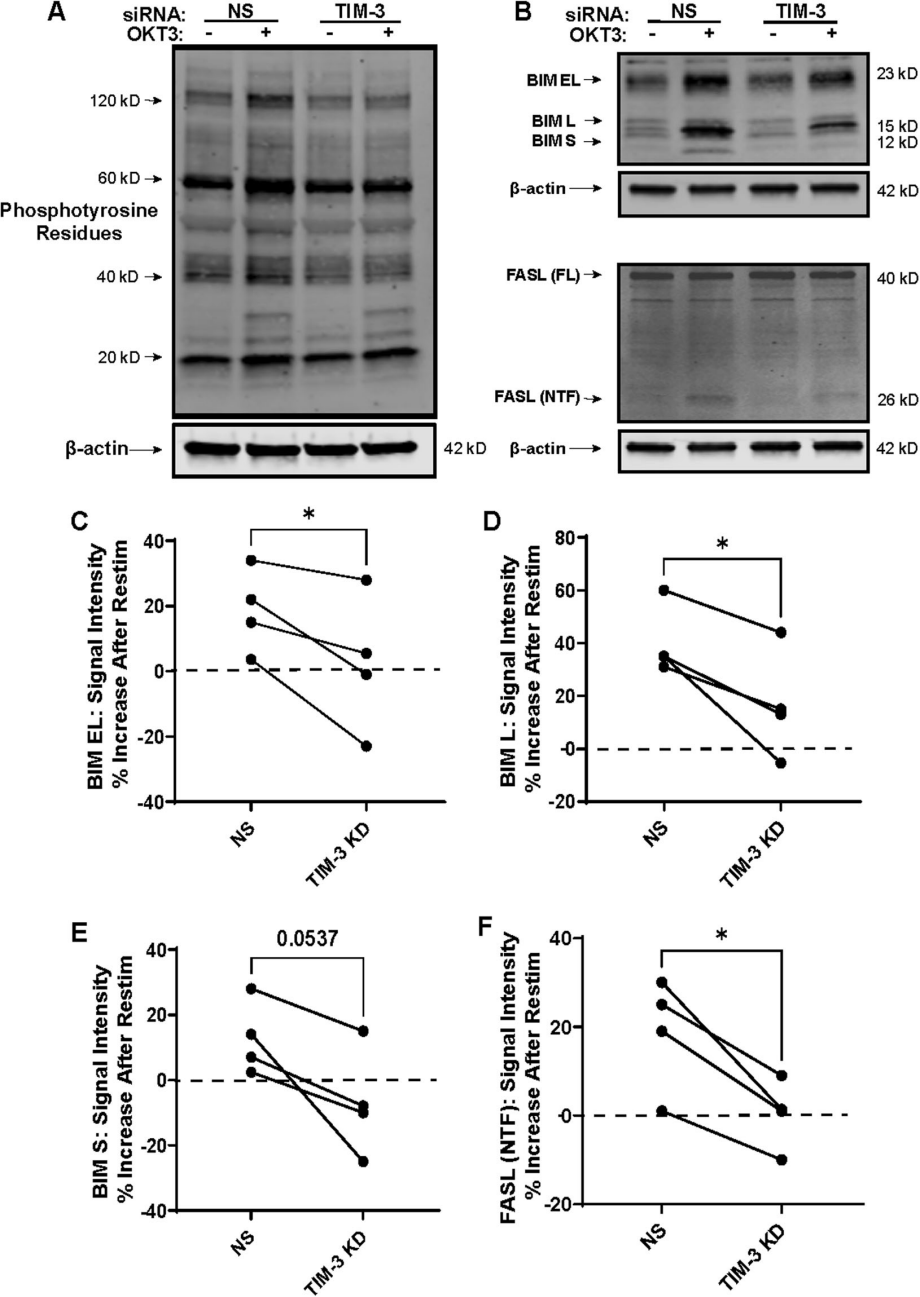
TIM-3 drives increased proximal TCR signaling and pro-apoptotic protein expression in late-stage effector CD8^+^ T cells. **A** Representative phosphotyrosine western blot of late-stage lysates following TIM-3 knockdown and restimulation with 100 ng/mL of OKT3. The blot represents 1 of 3 that were run from individual donors. **B** Western blots for BIM isoforms (EL = extra-long isoform, L = long isoform, S = short isoform) and Fas Ligand (FASL, FL = full-length, NTF = N-terminal fragment). Beta-actin serves as a loading control. **C**–**F** Differences in protein expression were assessed for statistical significance using spot densitometry, normalized to actin. Each connecting line represents a single donor. Asterisks denote statistical significance using the following paired *t* tests: **C** *p* = 0.0480, **D** *p* = 0.0265, **E** *p* = 0.0537, **F** *p* = 0.0157.

To investigate whether TIM-3 was influencing differential RICD sensitivity by changing proximal TCR signaling, we first assessed larger signaling changes following TCR restimulation. In late-stage effector T cells, TIM-3 knockdown decreased the magnitude of proximal TCR signaling as assessed by total phospho-tyrosine signal (Fig. 2A). Downstream, TIM-3 knockdown also decreased TCR restimulation-induced expression of pro-apoptotic proteins connected with RICD execution, BIM and FAS ligand (FAS-L)^32,33^ (Fig. 2B–F). Specifically, we noted a reduction in the cytoplasmic N-terminal fragment of FASL produced upon cell surface exposure and cleavage by matrix metalloproteinases, indicative of enhanced FASL surface expression. This was corroborated by reduced induction of FASL on the surface of TIM-3 KD cells by flow cytometry following restimulation (Supplementary Fig. 1C). Collectively, these results suggest that TIM-3 boosts proximal TCR signaling following restimulation in late-stage effectors, which facilitates the increased expression of proapoptotic proteins required for RICD, similar to the role played by SAP and NTB-A signaling^8^. In contrast, we observed no consistent differences in proximal signaling or pro-apoptotic protein expression following TIM-3 knockdown in newly activated cells (data not shown), suggesting that TIM-3 influences RICD protection during T cell expansion via other mechanisms.

### TIM-3 is expressed on the plasma membrane in early-stage effector T cells, but remains predominantly intracellular at later stages

**Fig. 3:**
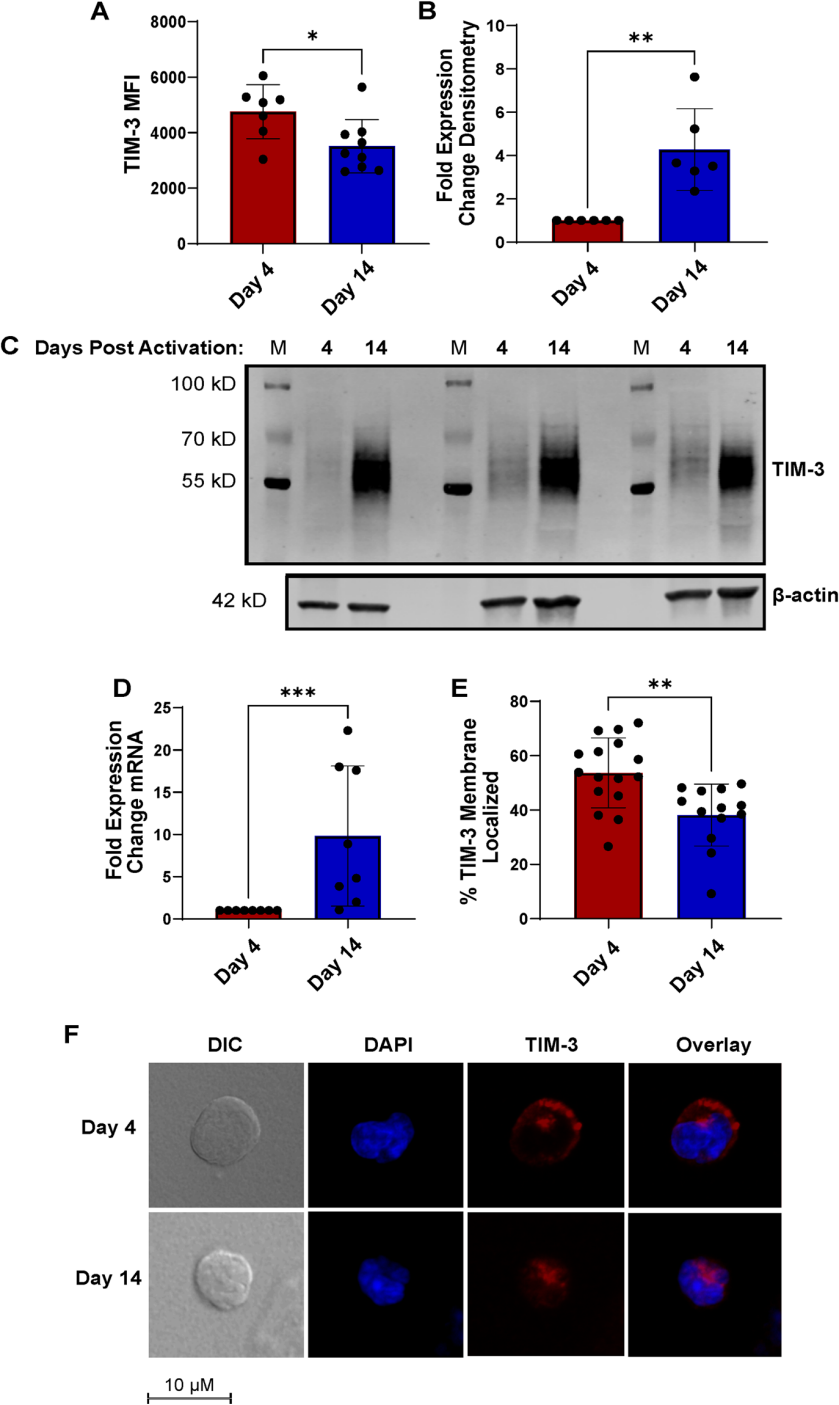
TIM-3 localization in effector CD8^+^ T cells changes over time in culture. **A** Surface TIM-3 expression (MFI) was measured by flow cytometry for early stage (day 4) and late-stage (day 14) effector T cells. **B** Total TIM-3 protein expression was quantified by spot densitometry of western blots, normalized against β-actin, plotted for each donor from early vs. late-stage T cells. **C** Representative western blot of three donors tested for total TIM-3 expression, as quantified in (**B**). M = marker. **D** TIM-3 mRNA expression was assessed by qPCR on day 4 and day 14 post-activation, with normalization against 18S rRNA. **E** Protein localization was quantified by confocal microscopy using ImageJ (see “Methods” section for code), in which 2–4 single cells from 6 individual donors (each point represents a representative cell from a different donor) on day 4 and day 14 were assessed for % TIM-3 distribution around the cell. **F** Representative images of single cells from days 4 and 14 post activation, as quantified in (**E**). Asterisks denote statistical significance using the following tests: **A** *t* test, *p* = 0.0229; **B** Mann–Whitney test, *p* = 0.0002; **D** Mann–Whitney test, *p* = 0.0022; **E** paired *t* test, *p* = 0.0125.

Based on the recent study of patients harboring germline mutations in *HAVCR2* (encoding TIM-3), differential localization of TIM-3 within the cell can influence function and pathology in patients with subcutaneous panniculitis-like T cell lymphoma (SPTCL)^34^. Therefore, we examined TIM-3 localization in newly activated versus late-stage effector T cells to potentially shed light on the mechanism underlying differential RICD sensitivity. We found that while TIM-3 surface expression is higher on day 4 than day 14 for each donor tested (Fig. 3A), TIM-3 total protein and mRNA expression was markedly higher in late-stage effectors (Fig. 3B–D). These data suggested that TIM-3 is primarily expressed on the plasma membrane immediately following activation, but remains predominantly intracellular in late-stage effector T cells. This finding was corroborated by microscopy analysis, which revealed more cell surface localization of TIM-3 in early-stage T cells, often in clusters (Fig. 3E, F). Collectively, these results suggest that differential RICD sensitivity could be driven by changes in localization of TIM-3.

### TIM-3 requires a ligand to protect newly activated cells from RICD

**Fig. 4:**
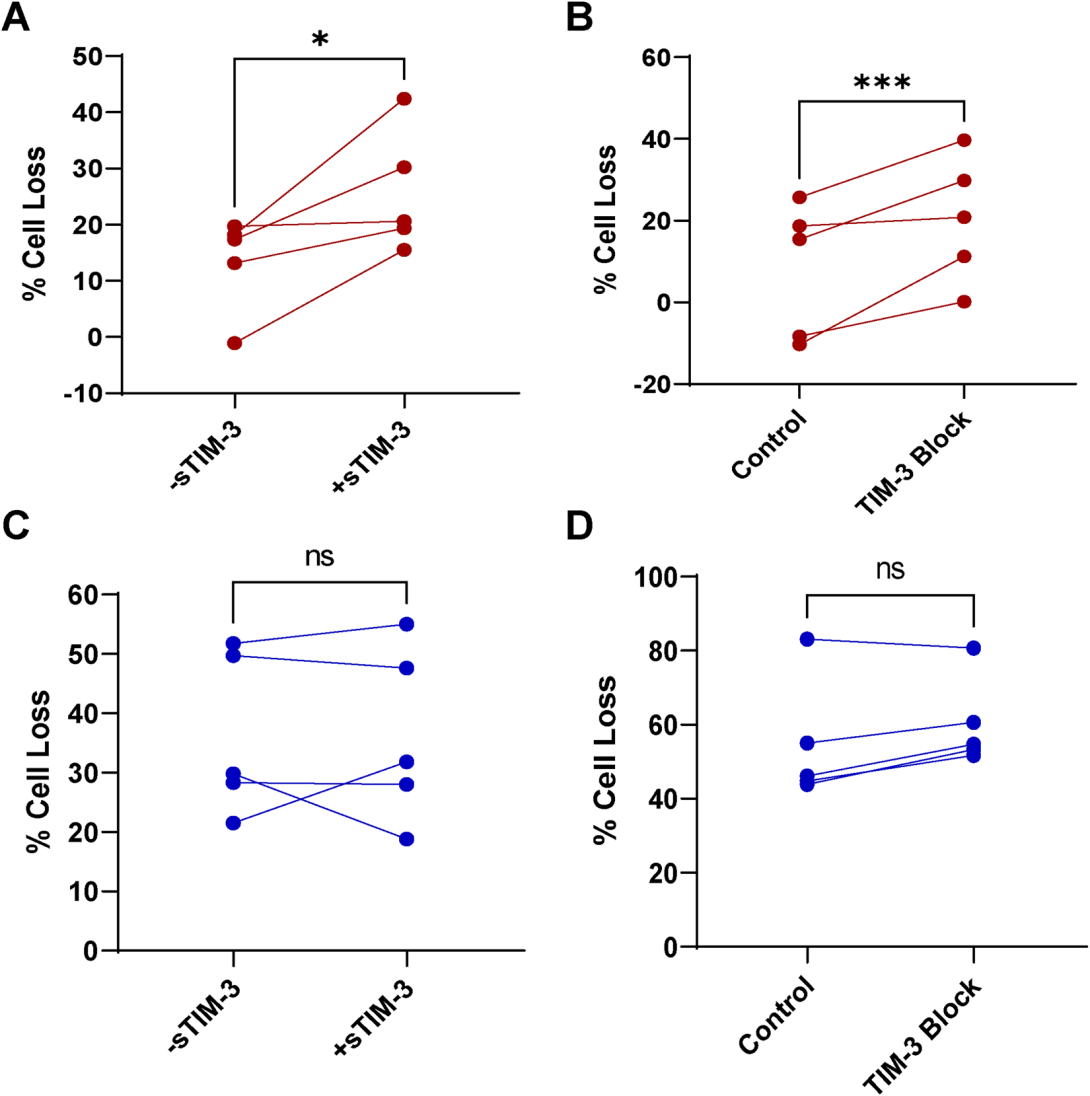
TIM-3 ligand binding is required for mitigating RICD sensitivity in early stage effector T cells. Effector T cells at day 4 (**A**) or day 14 (**C**) were treated with soluble TIM-3 (+sTIM-3) or isotype control (-sTIM-3) for 1 h prior to restimulation with 100 ng/mL of OKT3 and death assessment 24 h later. Cells were treated day 4 (**B**) or day 14 (**D**) with TIM-3 blocking antibody or an isotype antibody control for 1 h prior to restimulation with 100 ng/mL of OKT3 and death assessment 24 h later. Asterisks denote statistical significance using paired *t* tests: **A** *p* = 0.0399; **B** *p* = 0.0003; **C** *p* = 0.9957; **D** *p* = 0.0563.

Because TIM-3 is preferentially localized to the cell surface immediately following activation, we next asked whether a ligand was required for TIM-3-dependent protection from premature RICD at this stage. To investigate this, we added either soluble TIM-3 (Fig. 4A, C) or a TIM-3 blocking antibody (Fig. 4B, D) to newly activated (Fig. 4A, B) and late-stage (Fig. 4C, D) effector cells prior to restimulation and apoptosis assessment. Newly activated CD8^+^ T cells showed increased RICD sensitivity following the addition of soluble TIM-3 or TIM-3 blocking antibody, mirroring the effects of knocking down TIM-3 via siRNA at this stage. In contrast, these reagents had no effect on RICD sensitivity in late-stage effectors, a result consistent with TIM-3 intracellular localization. Collectively, these data imply that TIM-3 is dependent on a ligand to drive differential RICD sensitivity during clonal effector expansion. TIM-3 has a number of reported ligands^25,35–38^. Of the four identified ligands of TIM-3, three exhibit plasma membrane expression: phosphatidylserine (PS), Gal-9, and carcinoembryonic antigen-related cell adhesion molecule 1 (CEACAM1). Of these ligands, CEACAM1 showed the highest difference in surface expression by flow cytometry over time, mirroring the pattern of TIM-3 expression in effector T cells (Supplementary Fig. 1D). CEACAM1-4L has been documented to be the major isoform expressed; we independently verified this in our system using previously established isoform recognition primers^39^ (Supplementary Table 1). Intriguingly, the blocking antibody used in Fig. 4B, D has been shown to block the interaction of TIM-3 with CEACAM1, suggesting that CEACAM1 may be responsible for driving differential changes in RICD in conjunction with TIM-3 during clonal expansion^40^.

### Co-expression of TIM-3 and CEACAM1 increase TIM-3 surface expression in Jurkat T Cells

**Fig. 5:**
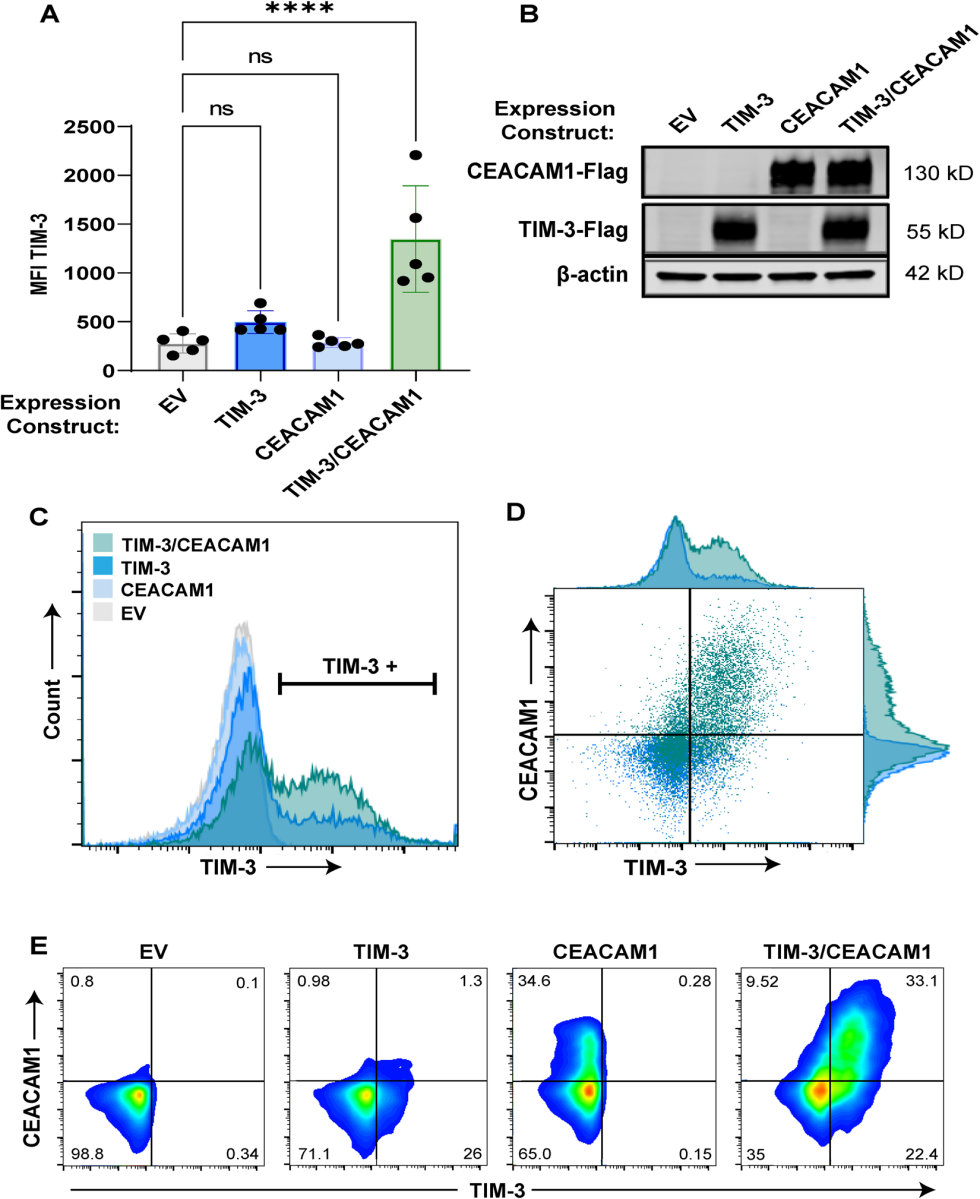
Co-expression of CEACAM1 promotes TIM-3 surface expression on T cells. **A** Jurkat T cells were transfected with empty vector (EV), TIM-3, CEACAM1, or TIM-3 and CEACAM1 FLAG-tagged expression plasmids and assessed for TIM-3 surface expression. Each point represents separate experiments in which the median fluorescence intensity (MFI) was assessed for each condition. Statistical tests: **A** one-way ANOVA with multiple comparisons; *p* < 0.0001 for EV against TIM-3/CEACAM1. **B** Cell lysates were assessed for total CEACAM1 and TIM-3 protein expression by western blot (anti-FLAG); β-actin served as a loading control. **C** Single experiment from (A) plotted as a histogram to highlight the increased % TIM-3^+^ cells with TIM-3 and CEACAM1 co-transfection. **D** Dot plot histogram overlay of Jurkats transfected with only TIM-3 (blue) versus both TIM-3 and CEACAM1 (teal). **D** Representative flow plots of Jurkats transfected as in (**A**).

It has been shown that CEACAM1 can effectively “chaperone” TIM-3 to the surface of transfected human embryonic kidney (HEK) 293T cells^38^; we therefore hypothesized that CEACAM1 also enables TIM-3 surface expression in human T cells. To further investigate the interaction of TIM-3 and CEACAM1, we employed transient ectopic expression in Jurkat T cells, which lack endogenous expression of both proteins. We found that TIM-3 was markedly increased on the cell surface when CEACAM1 was co-expressed, despite similar total protein expression across all conditions (Fig. 5A, B). Not only was the median fluorescence intensity of TIM-3 and CEACAM1 higher with co-expression, but the total proportion of TIM-3^+^ cells was also markedly higher, indicating that CEACAM1 supports TIM-3 surface expression in T cells (Fig. 5C–E). Although transformed Jurkat T cells are generally unsuitable for RICD analyses, these data confirm that CEACAM1 can escort TIM-3 to the surface of activated T cells, suggesting CEACAM1 may be implicated in changing RICD sensitivity of primary CD8^+^ T cells over time due to its influence over TIM-3 cellular localization. Although Gal-9 has also been tested as an apoptotic factor in Jurkat cells^41,42^, we did not test whether it or PS influenced TIM-3 expression or RICD function in our cultures.

### TIM-3 surface localization is dependent on CEACAM1 in primary T cells

**Fig. 6:**
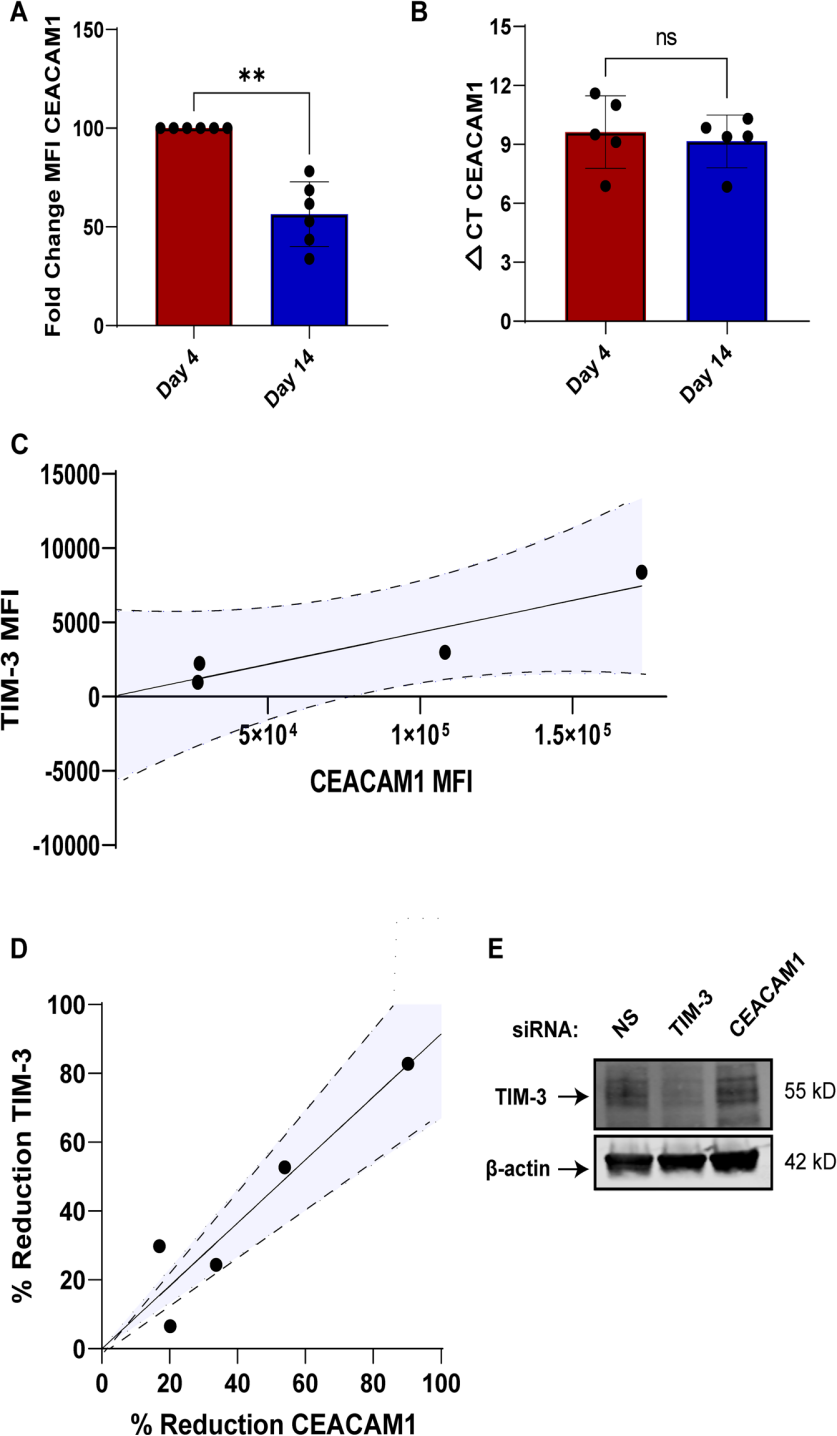
TIM-3 cellular localization is dependent on CEACAM1 in primary CD8^+^ effector T cells. **A** CEACAM1 was assessed by flow cytometry (MFI) on days 4 and 14 post-activation; data normalized to day 4. **B** CEACAM1 mRNA was collected on days 4 and 14 post-activation and assessed by qPCR, normalized to 18S rRNA, and plotted as ΔCt values. **C** CEACAM1 and TIM-3 MFI were plotted for individual donors on day 4 post-activation. Dotted lines represent 95% confidence intervals. **D** Cells from five donors transfected with CEACAM1 siRNA on day 0 were assessed for both CEACAM1 and TIM-3 expression on day 4. Dotted lines represent 95% confidence intervals. **E** Representative western blot (of three donor replicates) probing for TIM-3 following either TIM-3 or CEACAM1 knockdown in newly activated cells (day 4). Statistical tests: **A** Mann–Whitney test, *p* = 0.0022; **B** paired *t* test, *p* = 0.6575; **C** simple linear regression and Pearson correlation, *p* = 0.0392; **D** simple linear regression and Pearson correlation, *p* = 0.0172.

To verify the ability of CEACAM1 to change TIM-3 localization in primary T cells, we investigated CEACAM1 expression first independently and then in conjunction with TIM-3. We found that while CEACAM1 surface expression mirrored that of TIM-3 over time in culture (Fig. 6A), CEACAM1 total mRNA expression remained steady (Fig. 6B). TIM-3 surface expression was also closely correlated with CEACAM1 surface expression for multiple donors (Fig. 6C). Intriguingly, CEACAM1 knockdown decreased TIM-3 surface expression without affecting total TIM-3 protein levels in early-stage effector T cells (Fig. 6D, E). These results collectively suggest that CEACAM1 drives TIM-3 localization not only in immortalized cell lines, but also in primary T cells as well.

### TIM-3 and CEACAM1 work together to protect newly activated cells from premature RICD

**Fig. 7:**
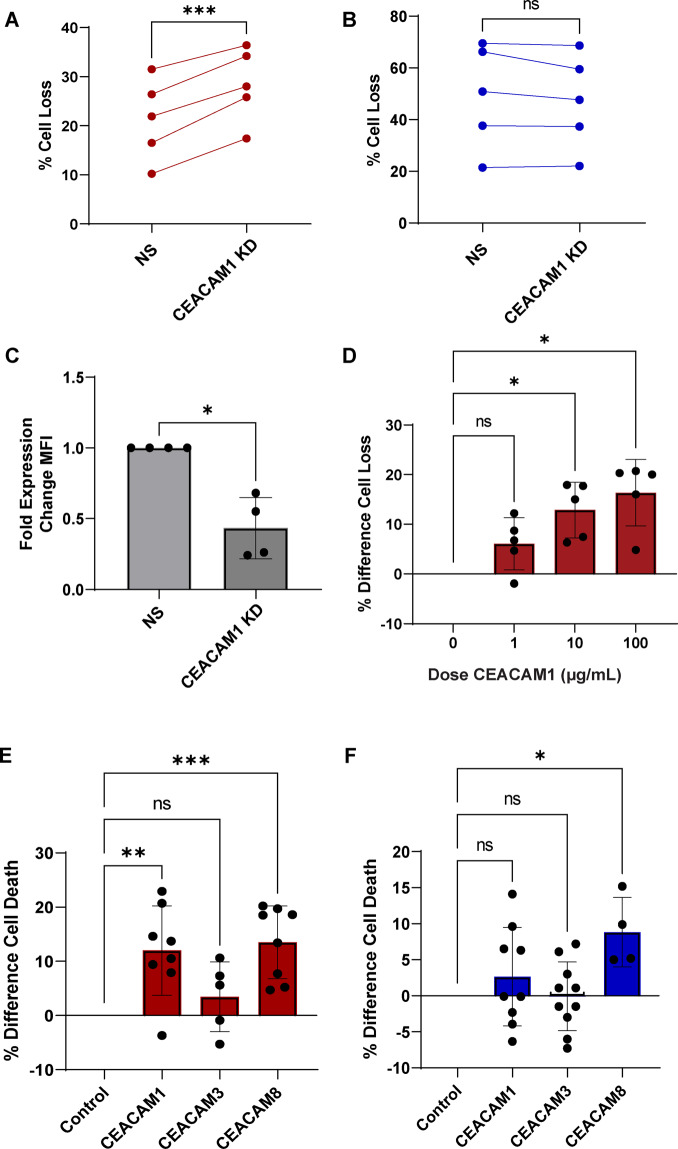
TIM-3 and CEACAM1 cooperate to protect newly activated cells from RICD. **A** T cells transfected with either CEACAM1-specific siRNA (CEACAM1 KD) or nonspecific siRNA (NS) were assessed for RICD sensitivity on day 4 post-activation as previously mentioned. **B** Cells transfected with siRNA as in (**A**) on day 10 were assessed for RICD on Day 14 post-activation. **C** CEACAM1 KD efficiency was evaluated using flow cytometry, plotted as fold decrease for individual experiments. **D** Newly activated cells were treated with increasing doses of CEACAM1-Fc chimera for 1 h prior to restimulation and assessed for apoptosis 24 h later using propidium iodide staining and flow cytometry. **E** Newly activated cells were treated with CEACAM1-Fc, CEACAM3- Fc, or CEACAM8-Fc chimera protein, or solvent control on Day 4 post-transfection and assessed for RICD sensitivity following OKT3 restimulation. **F** Late-stage effector cells (Day 14) were treated as in (**E**), dose ranging from 10 to 100 µg/mL. Asterisks denote statistical significance using the following tests: **A** paired *t* test, *p* = 0.0007; **B** paired *t* test, *p* = 0.1843; **C** Mann–Whitney test, *p* = 0.0286; **D** repeated measures one-way ANOVA, control vs. 1 µg/mL, *p* = 0.1312; control vs. 10 µg/mL, *p* = 0.0156, control vs. 100 µg/mL, *p* = 0.0127; **E** ordinary one-way ANOVA with multiple comparisons (corrected), control vs. CEACAM1 *p* = 0.0016; control vs. CEACAM3 *p* = 0.5216; control vs. CEACAM8 *p* = 0.0006 **F** ordinary one-way ANOVA with multiple comparisons (corrected), control vs. CEACAM1 *p* = 0.44; control vs. CEACAM3 *p* = 0.9991; control vs. CEACAM8 *p* = 0.05.

To investigate whether CEACAM1 was driving differential RICD sensitivity in conjunction with TIM-3, we first asked whether CEACAM1 influences RICD sensitivity in effector T cells on its own. We found that CEACAM1 siRNA-mediated knockdown increased RICD in newly activated cells, but had no effect on RICD sensitivity in late-stage effectors (Fig. 7A, B). This pattern of CEACAM1-dependent protection from premature RICD mirrors the effect of TIM-3 in newly activated cells (Fig. 1B), supporting the hypothesis that they work together to protect new effector T cells from premature RICD during clonal expansion. Administering increasing doses of recombinant CEACAM1 during clonal expansion resulted in modest but significant increases in RICD sensitivity, implying the dependence of RICD sensitivity on endogenous TIM3-CEACAM1 co-expression at this critical stage (Fig. 7D). Addition of CEACAM8 (which binds to CEACAM1 and 6) also increased RICD in newly activated cells, whereas soluble CEACAM3 (which does not bind to CEACAM1^43,44^) had no significant effect (Fig. 7E). Intriguingly, neither CEACAM1 nor CEACAM3 changed RICD sensitivity in late-stage effectors, further implying that the interaction of TIM-3 and CEACAM1 on the plasma membrane is crucial to providing RICD protection only during clonal expansion (Fig. 7F). Interestingly, CEACAM8 administration did increase RICD slightly (Fig. 7F), suggesting the broader family of CEACAM molecules is worthy of further investigation in this process. From these data, we surmise that CEACAM1 promotes TIM-3 surface localization, and that they work as a pair to facilitate protection from premature RICD in early-stage effector CD8^+^ T cells.

## Discussion

In this study, we reveal a novel role for TIM-3 as a key regulator of RICD in CD8^+^ effector T cells. Surprisingly, our findings indicate that TIM-3 affects RICD differently at distinct timepoints following T cell activation. In newly activated CD8^+^ effector T cells, TIM-3 partners with CEACAM1 to shield them from premature RICD, helping to ensure proper clonal expansion. For late-stage, terminally differentiated effector T cells, however, TIM-3 potentiates RICD by enhancing TCR signal strength. Our findings suggest that RICD resistance is driven in part by CEACAM1 “escorting” TIM-3 to the plasma membrane early after initial TCR stimulation; an effect lost in late-stage effectors wherein TIM-3 remains largely intracellular.

Ultimately, our results demonstrate that TIM-3 helps to regulate RICD sensitivity of effector CD8^+^ T cells. To this point, TIM-3 has most thoroughly been characterized as an exhaustion marker on tumor-infiltrating T lymphocytes. Indeed, severely exhausted cells are marked by elevated TIM-3 expression^12,30,45,46^. However, collective studies of TIM-3 have highlighted considerable functional variability in different contexts. Kane and colleagues first described the ability of TIM-3 to enhance TCR signaling in Jurkat T cells^47^. These findings appear contradictory to the body of literature suggesting that TIM-3 inhibits T cell responses^16,20,28,48^, coincident with the hypothesis that TIM-3 contributes to exhaustion. However, recent work from Dr. Rafi Ahmed and others has demonstrated that TIM-3^+^ CD8^+^ cells in chronic viral infections are severely exhausted and beyond the rescue of anti-PD-1/PD-L1 checkpoint blockade, considering TIM-3 blockade alone is insufficient to rescue these cells from senescence and apoptosis commitment^29,81^. We posit that these late-stage “terminally exhausted” cells, in which TIM-3 may be primarily intracellularly localized, could be accelerated towards their death through TIM-3-enhanced RICD, though this requires further investigation. Our data therefore helps to clarify some of these conflicting points in the literature, and highlights a novel role for TIM-3 as a key regulator of normal CD8^+^ T cell homeostasis.

Our work further emphasizes the nuances of TIM-3 function and the role that interaction with specific ligands plays in defining its functions. We show that TIM-3 can augment TCR signaling following restimulation in late-stage effector T cells, leading to increases in downstream pro-apoptotic protein expression; this is congruent with the aforementioned study by Lee et al.^47^. It is important to note that in that study, TIM-3 ligands were not necessary for driving differences in signaling output, a finding which is corroborated by our data^47^. Downstream of these events, TIM-3 has been documented to promote PI-3K and AKT signaling^49,50^. We found that while treating newly activated cells with PI-3K and AKT inhibitors had no appreciable effect on RICD, late-stage effectors showed decreased RICD following inhibitor treatment (Supplementary Fig. 2A, B). Further work will be needed to determine whether intracellular TIM-3 is facilitating increased proximal PI-3K and AKT signaling as part of its role in potentiating RICD. Moreover, downstream effects on the expression of other pro- (e.g., NUR77 and NOR1^9^) and anti-apoptotic proteins (e.g., BCL-xL^51^) beyond FASL and BIM should also be assessed. Remarkably, previous microarray analyses indicate that TIM-3 expression is also dependent on SAP in this late stage^3^, implying that reduced TIM-3 levels (~1.8-fold) may contribute to reduced pro-apoptotic protein induction and RICD resistance in SAP-deficient CD8^+^ effector T cells from XLP-1 patients.

In newly activated T cells, the mechanism by which TIM-3 and CEACAM1 protect cells from premature RICD also remains unresolved. CEACAM1 harbors a cytoplasmic immunotyrosine inhibitory motif (ITIM) for phosphastase recruitment, working to dampen the strength of the TCR signal^52^. On the other hand, TIM-3 ligand engagement is known to displace Bat3, leaving LCK more vulnerable to dephosphorylation and diminished proximal TCR signaling^53^. We found that silencing TIM-3 during initial T cell activation did not have a measurable impact on proximal TCR signaling (data not shown), although more detailed studies are warranted. One could hypothesize that TIM-3 is needed on the surface merely as a ligand for CEACAM1, which is dampening proximal signaling via its ITIM alone. However, addition of recombinant CEACAM1 enhanced RICD, ostensibly by outcompeting TIM-3-CEACAM1 interactions. These data imply that CEACAM1 engagement via homotypic interaction does not confer RICD protection, suggesting that surface TIM-3 and CEACAM1 must cooperate to enforce RICD resistance. Although other studies have suggested that TIM-3 and CEACAM1 do not interact^54^, our study bolsters the argument that CEACAM1 plays a central role in TIM-3 regulation in CD8^+^ effector T cells, consistent with the original interaction characterized by Huang et al.^38^. Indeed, CEACAM1 knockdown changed TIM-3 surface localization but not total expression in early stage effectors (Fig. 6), making it plausible that increased TIM-3 intracellular localization in newly activated cells forced them to behave more like late-stage effectors with elevated RICD sensitivity. Additional experimentation will be required to ascertain the proportional inputs of TIM-3 and CEACAM1, both individually and together, in fine-tuning RICD sensitivity over time. Our results also indicate that TIM-3 works with CEACAM1 to facilitate resistance to RICD in newly activated cells. Although our data suggest this effect is mediated via coupled TIM-3-CEACAM1 surface expression, further work will be needed to distinguish whether this phenomenon requires any other extracellular autocrine ligands. Despite these unknowns, our data indicate that TIM-3 function in human CD8^+^ T cells is intimately tied to its temporal expression, cellular localization, and ligand availability.

CEACAM1 is a member of the carcinoembryonic-antigen-related cell adhesion molecule family, known to be involved in influencing a wide number of normal cellular processes through both hetero- and homophilic interactions^55^. The isoform predominantly expressed in T cells—CEACAM1-4L—has a long cytoplasmic tail containing ITIM motifs which dampen proximal TCR signaling by targeting ZAP70^52^. Because of its status as a coinhibitory protein, CEACAM1 has been tested as a potential therapeutic target alongside other checkpoint blockade regimens, with some success^56–59^. However, its function with regard to programmed cell death is unclear. One study found that CEACAM1 expression in Jurkat T cells prevented FAS-mediated apoptosis by directly redistributing beta-catenin in the actin cytoskeleton^60^, whereas another study by Nittka et al.^61^ described that CEACAM1 can promote apoptosis in Jurkat cells through direct activation of caspases. Much like TIM-3, the cellular context within which CEACAM1 is studied is likely important for understanding its full functional capacities within the immune system. Our study demonstrates a novel function for CEACAM1 in protecting expanding cells from premature RICD, working in concert with TIM-3. This finding also sheds new light on patients with specific germline TIM-3 mutations, a significant proportion of whom suffer from SPTCL^34,62,63^. In these patients, TIM-3 cannot localize to the plasma membrane, which the authors noted led to “persistent immune activation and increased production of inflammatory cytokines… promoting SPTCL”^34^. Indeed, TIM-3 mutations noted in SPTCL are predicted to disrupt CEACAM1 association (data not shown). These results are consistent with our findings in late-stage effectors, in which TIM-3^+^ lymphocytes have increased proximal T cell signaling output. We predict that although more effector CD8^+^ T cells may undergo RICD at early stages, a pool of over-activated clones lacking TIM-3/CEACAM1-dependent signal modulation can drive damaging immunopathology in the form of SPTCL. In all, these results suggest that TIM-3 and CEACAM1 must work cooperatively to maintain immune system homeostasis via RICD regulation.

Finally, this study has revealed that coinhibitory proteins like TIM-3 can fine-tune RICD sensitivity in effector CD8^+^ T cells. Early studies offered conflicting results on how CD28 co-stimulation and CTLA-4 expression may influence activation-induced cell death/RICD, particularly in CD4^+^ T cells^64–67^. RICD has long been recognized as a key homeostatic process for constraining effector T cell expansion^2^. Several factors govern RICD sensitivity in late-stage effector T cells, but little is known about mechanisms of RICD resistance in early expanding T cells. A recent study from our group showed that early expanding conventional human effector T cells are less susceptible to RICD in part due to the influence of the transcription factor FOXP3 and autophagy^10^. It is also plausible that newly activated T cells avoid RICD via downregulation of TCR/CD3 expression^68^. Indeed, we have found that effector CD8^+^ T cells do express less CD3 at early versus late timepoints after activation (Supp. Fig. 2C). However, early effector T cells also express higher levels of CD25 after stimulation (Supplementary Fig. 2D), which presumably enhances RICD sensitivity via increased IL-2 responsiveness and cell proliferation^5^. Intriguingly, CEACAM1 has also been documented to decrease IL-2 production in expanding T lymphocytes, which may act to keep clonal expansion and RICD sensitivity in check despite high CD25 expression^69^. Because TIM-3 and CEACAM1 work together to protect newly activated cells from RICD, our results underscore a mechanism by which expanding CD8^+^ T cells modulate responses (including apoptosis) by fine-tuning TCR signal strength via co-inhibitory proteins. Indeed, our results corroborate the “Tide Model” of T cell immunity, in which dynamic, tightly regulated expression of costimulatory and coinhibitory proteins dictate T cell fate and ensure optimal control of population expansion and contraction^70,71^. Our results suggest this process also governs RICD sensitivity in CD8^+^ T cells, whereby signal calibration via coinhibitory and costimulatory proteins helps to determine their temporal susceptibility to cell death in response to TCR re-engagement. Future studies should expound upon this hypothesis to better understand how other co-inhibitory proteins can shape the overall T cell response by promoting RICD resistance, particularly during clonal expansion. Moreover, it may also be important to delineate how co-signaling proteins like TIM-3 influence RICD in effectors derived from naïve and various memory CD8^+^ T cell subsets.

In conclusion, this study describes a novel function for the coinhibitory proteins TIM-3 and CEACAM1 in mitigating RICD in expanding effector CD8^+^ T lymphocytes during clonal expansion. In contrast, TIM-3 works independently of CEACAM1 to potentiate RICD in late-stage effector T cells. This study implies that the utilization of checkpoint blockade antibodies may have broader effects on the immune response than previously appreciated, including dysregulation of cell death sensitivity. In fact, a recent report showed loss of CEACAM1 leaves mice more susceptible to enteropathogenic bacterial infection, specifically through a dysregulated, hyperactive CD8^+^ T cell response in the colon^72^. These concepts may warrant further consideration for those studying adverse outcomes and variable efficacy associated with checkpoint blockade regimens^73–77^, with the goal of specifically reinvigorating exhausted cells without inducing overt immunopathology or compromising nascent T cell responses.

## Materials and methods

### Blood collection and effector T cell selection

Peripheral blood mononuclear cells (PBMC) were acquired from healthy human volunteers at the National Institutes of Health Blood Bank (access kindly provided by Dr. Michael Lenardo). PBMC were separated using a Ficoll separation gradient. Following centrifugation, the PBMC layer was extracted and washed twice using phosphate-buffered saline (PBS). CD8^+^ T cells were isolated via negative selection using Stem Cell Magnetic Bead Separation Kit (17953, Stem Cell Technologies, Vancouver, Canada) according to the manufacturer’s instructions.

### Cell culture and activation

Cells were activated using Immunocult Human CD3/CD28/CD2 T Cell Activator (Stem Cell Technologies, #10970) and cultured in RPMI 1640 media (RPMI 1640 ThermoFisher Scientific, Waltham, MA) supplemented with 2 mM l-glutamine, 10% fetal bovine serum (FBS) (Millipore Sigma, Burlington, MA) and an antibiotic cocktail of 1% penicillin and streptomycin (Lonza, Basel, Switzerland). On day three post activation, cells were washed in PBS and cultured in complete RPMI media supplemented with 200 U/mL recombinant human IL-2 (#100-02, Peprotech, Rocky Hill, NJ). Primary cells were cultured for up to 20 days post activation, checked daily for cell count, and kept at a concentration between 1 and 2 × 10^6^ cells/mL. Jurkat T cells (ATCC clone E6.1) were maintained in the above media conditions as well without supplemental IL-2.

### siRNA transfection

Cells were transfected with 250–500 pmol TIM-3 siRNA (ThermoFisher Scientific, 4392420), CEACAM1 siRNA (Integrated DNA Technologies (IDT), Coralville, IA) or nonspecific siRNA scramble control (Thermo Fisher, 12935300) (see Supplemental Table 1 for siRNA sequences) using a P3 kit for the Amaxa 4D Nucleofector (Lonza). All cells were assessed for knockdown efficiency by quantitative reverse transcription polymerase chain reaction (qRT-PCR), flow cytometry or western blotting on days 3–4 post-transfection. Freshly isolated T cells were transfected immediately post negative selection, allowed to rest for 30 min, and activated with Immunocult (Stem Cell Technologies) according to the manufacturer’s instructions. Late-stage T cells (days 10–14 post-activation) were transfected on day 10 and assessed for knockdown efficiency on day 13 post-activation.

### Pharmacological Inhibitors

Cells were treated with 1 µM AKT inhibitor AZK5363 (S8019) or 1 µM PI3K inhibitor BKM120 (S2247), Selleck Chemicals, Houston, TX), or DMSO solvent control for 1 h prior to restimulation with 100 ng/mL OKT3. Cells were assessed for death 24 h later via propidium iodide staining and flow cytometry as detailed below.

### Cloning and cell transfections

TIM-3 and CEACAM1 DNA constructs were purchased from Addgene (49212, Watertown, MA) and Origene (RC221096, Rockville, MD), respectively. Open reading frames were cloned into the pUNO expression vector using NEBuilder HiFi DNA Assembly kit (New England Biolabs, Ipswich, MA), including a C-terminal FLAG tag. Purified plasmids (10 µg per cuvette) were transfected into Jurkat T cells using a ECM630 electroporator (Harvard Apparatus BTX, Holliston, MA) at 260 V, 950 mF as previously described^78^. Expression of proteins was assessed by flow cytometry or western blot.

### RICD assays

Primary effector T cells in RPMI were aliquoted into 96 well plates at 1 × 10^6^ cells/mL and restimulated with anti-CD3 antibody OKT3 (#05121-05, Bio-gems, Westlake Village, CA). Unless otherwise stated, all experiments performed per independent blood sample were completed with three technical replicates. All restimulation experiments were performed using 100 ng/mL of OKT3 unless otherwise stated. For death assessment, cells were cultured for 24 h in OKT3 and assessed for apoptosis 24 h later using propidium iodide staining and flow cytometry (see below). For newly activated cells, cells were restimulated on day 4 post-activation; late-stage cells were restimulated on day 13 post-activation.

### Flow cytometry

#### General flow cytometry protocol

T cells were spun in polypropylene round-bottom tubes and placed in 100 µL of fluorescence-activated cell sorting (FACS) buffer (PBS supplemented with 0.1% sodium azide, 1% FBS). Tubes were stained with antibodies, vortexed briefly and placed on ice in the dark for 30 min. Following antibody binding, tubes were washed twice with 2 mL FACS buffer and spun down. Prior to flow cytometric analysis, cells were resuspended in 300 µL FACS buffer and immediately analyzed on an Accuri C6 flow cytometer (Becton Dickinson, Franklin Lakes, NJ). TIM-3 surface staining was performed using PE-conjugated anti-TIM-3 antibody (clone F38-2E2, Biolegend, San Diego, CA) at 5 µL/test; CEACAM1 surface staining was performed using unconjugated anti-CEACAM1 monoclonal antibody (clone 283340, ThermoFisher Scientific) with secondary antibody (Biolegend, 406715), both at 2 µL/test. APC-conjugated anti-CD3 (100236), PE-conjugated anti-CD25 (356104), and PE-conjugated anti-FASL (306407) antibodies were used at 5 µL/test (Biolegend). TIM-3 ligands were assessed as follows: anti-Gal-9 antibody (clone 9M1-3, Biolegend, San Diego, CA) at 5 µL/test; anti-PS antibody (Apotracker Green, Biolegend, San Diego, CA).

#### Time course assessment

Cells were assessed on days 0, 2, 4, 7, 10, 13, 16, and 19 following activation for TIM-3 (clone F38-2E2, 345006) at 5 µL/test.

#### Propidium iodide staining

Following overnight restimulation, cells were collected from the 96-well plate and placed into 0.3 ml polypropylene tubes. Ten microlitre of propidium iodide (PI) (Millipore Sigma, P4864) at 1 µg/mL stock solution was added to each tube. Cells were immediately assessed for apoptosis by flow cytometric analysis on the BD Accuri C6.

#### Flow cytometric software

All flow cytometric analyses were performed using Accuri C6 Flow Cytometer built-in software and FlowJo (Becton Dickinson).

### Addition of blocking antibodies and recombinant proteins

To disrupt TIM-3 ligand association, primary effector T cells were cultured with 10 µg/mL TIM-3 blocking monoclonal antibody (clone F38-2E2, Biolegend, 345004), Recombinant Human TIM-3-FC Chimera (R&D Systems, 2365-TM-050), or 10 µg/mL Mouse IgG1 Isotype Control SAFIRE (Bio-gems, 44212-25) for 1 h prior to restimulation with OKT3. CEACAM1, CEACAM3 and CEACAM-8 Fc-chimera proteins were generated from plasmids kindly provided by Dr. Esther Klaile (University of Jena, Germany) as previously described^79^, and were used at a concentrations between 1 and 100 µg/mL in primary cell solution for 1 h prior to restimulation with OKT3, as noted in the text. CEACAMx-Fc plasmids were originally synthesized in the labs of Esther Klaile and Bernard Singer.

### Calculation of RICD

RICD sensitivity was calculated as previously described^80^. Propidium iodide-negative cells (viable) were counted for each sample under a fixed rate of fluidics and time of collection on the AccurI C6 Flow Cytometer. The following formula was used to calculate percentage cell loss: [% Cell Loss = ((Control − Restim)/Control)*100], for which the Control equaled the total number of untreated viable cells, and Restim equaled the total number of viable cells following restimulation with OKT3. Triplicate wells were averaged prior to assessment.

### Immunoblotting and densitometry analysis

#### Immunoblotting

Cells in each condition were lysed in 1% Nonidet P-40 (NP-40) lysis buffer (50 mM Tris [pH 7.4], 150 mM NaCl, 0.5 mM EDTA, 1% NP-40, 0.5% sodium deoxycholate, 1 mM Na_3_VO_4_, and 1 mM NaF), supplemented with protease inhibitors (Sigma, 11836153001) and PhosStop Phosphatase Inhibitors (Sigma, 4906837001). Cell lysates were mixed with in a 1:1 ratio of Laemmli buffer supplemented with 50 µM βME and heated at 95 °C for five minutes for denaturation. Proteins were separated on a 4–20% gradient acrylamide gel (456–1095, Bio-Rad, Hercules, CA) at 110 V for 90 min for optimal band separation. Gels were transferred to a nitrocellulose membrane via semi-wet transfer (TurboBlot tranfer system, Bio-Rad). The following primary antibodies were used at dilutions in Odyssey blocking buffer TBS (927-50000, LiCOR, Lincoln, NE) according to manufacturer’s protocol: TIM-3 XP monoclonal antibody (clone D5D5R, Cell Signaling Technology, 45208S), 4G10 Platinum Anti-Phosphotyrosine Antibody (Millipore Sigma, 2859168), BIM/BOD polyclonal antibody (ADI-AAP-330E, Enzo, Farmingdale, NY), FasL monoclonal antibody (clone: Ab3, EMD Biosciences, Millipore Sigma), anti-FLAG monoclonal antibody (Sigma, F1804-200UG), β-actin monoclonal antibody (Sigma, A1978). Licor IRDye secondary antibodies used at 1:10,000 dilution. All immunoblot images were visualized using an Odyssey CLx instrument (LiCOR) and assessed for densitometry using Image Studio Lite Software. To calculate densitometry, total band signal density was assessed in comparison to total β-actin signal density for normalization.

### Quantitative real-time PCR

Cells in each condition were prepared for RNA isolation according to manufacturer’s instructions (kit #11-331, Zymo Research, Irvine, CA). RNA was resuspended in nuclease free water at a concentration of approximately 50–100 ng/µL per sample. RNA samples were reverse transcribed into cDNA using 50–100 ng RNA total, using the iScript Advanced cDNA Synthesis Kit (Bio-Rad, 1725038). qRT-PCR was performed using the SSO Advanced SYBR Green qPCR Master Mix and manufacturer’s protocol (Bio-Rad, 1725271); qRT-PCR was performed on Bio-Rad CFX96. Primer sequences are listed in Supplemental Table 1. All samples’ Ct values were normalized to 18S Ribosomal RNA.

### Confocal microscopy

#### Slide preparation

Approximately, 30–100 × 10^3^ cells were placed onto poly-l-lysine-coated slides (Electron Microscopy Sciences (EMS), Hatfield, PA) at a concentration of 1 × 10^6^ cells/mL in PBS. All cells were fixed in 2% paraformaldehyde (EMS). Cells were washed with PBS and permeabilized using 0.1% Triton in PBS for 5 min at room temperature and subsequently washed with PBS. Cells were blocked using a 1% FBS/PBS mix for 30 min, followed by another PBS wash. Primary TIM-3 antibody (clone F38.2E2, Biolegend, 345004) was prepared in 1% PBS/FBS at a final concentration of 1 µg/mL and allowed to bind to cells for 30 min. Cells were washed three times with PBS/FBS solution. AlexaFluor-647 anti-IgG2b secondary antibody (Biolegend, 406715) was prepared in 1% PBS/FBS at a final concentration of 1 µg/mL and allowed to bind for 30 min. Cells were washed again with PBS/FBS three times. Ten microlitre DAPI Fluoromount-G mounting medium (0100-20, Southern Biotech, Birmingham, AL) was applied to the slides for DAPI staining and coverslip adhesion. All images were obtained on a Zeiss 700 confocal scanning microscope.

#### Image processing/code availability

Microscopy analyses were conducted using ImageJ (Fiji) software version 1.53c. Image processing code can be found on Github using the following link: mad-scientist-in-training/Microscopy-code: A repository for the code used in ImageJ to process the images present in the publication “TIM-3 drives temporal differences in RICD Sensitivity in conjunction with CEACAM1 in effector T lymphocytes” (github.com).

### Statistical analysis

All statistical analyses were performed and visualized using Prism software version 8.4.3 (GraphPad, San Diego, CA). Bar graph heights represent sample means, and all error bars indicate the standard deviation within the samples. For specific statistical tests, please reference the figure legends. All tests in which data was transformed significantly (including qPCR results and fold change values) were analyzed using the non-parametric equivalent of the appropriate statistical test to minimize type I error. All statistical tests were run two-sided. All *p* values < 0.05 were considered significant.

## Supplementary information

Supplemental Figure Legends

Supplemental Figure 1

Supplemental Figure 2

Supplemental Table 1

## Data Availability

Datasets not included in this article are available from the corresponding author on request.
