# Supplementary material for: TIM-3 drives temporal differences in restimulation-induced cell death sensitivity in effector CD8+ T cells in conjunction with CEACAM1
Source: Cell Death Dis. 2021 Apr 14;12(4):400. doi: 10.1038/s41419-021-03689-6 (PMC8046753; doi:10.1038/s41419-021-03689-6)
Supplement: Supplementary file 1 — Supplemental Figure Legends [file 41419_2021_3689_MOESM1_ESM.docx]

**Supplemental Figure Legends**

**Supplemental Table 1:** Primers and oligo sequences used in this study.

**Supplemental Figure 1: (A)** CD8+ T cells were assessed for TIM-3 knockdown efficiency following siRNA transfection. Representative histogram displays single donor TIM-3 MFI late-stage. **(B)** CD8+ T cells were assessed for TIM-3 expression on Day 4 and Day 14 post-activation by flow cytometry. Representative histogram overlay of a single donor with both time points. **(C)** Late-stage effector T cells were transfected with TIM-3-specific siRNA (TIM-3 KD) or nonspecific siRNA control (NS) and restimulated for 4 hours with 100 ng/mL OKT3. Cells were assessed for FASL expression by flow cytometry, and % increase in FASL MFI from baseline was calculated per donor (represented by connecting lines). **(D)** CD8+ T cells were assessed on Day 4 (red) and Day 14 (blue) for TIM-3 ligand expression by flow cytometry; the MFI was quantified and plotted. A total of 4 donors were tested for each ligand.

**Supplemental Figure 2: (A)** Newly activated (day 4) or **(B)** late-stage effector CD8^+^ cells were treated with 1 µM AKT inhibitor AZD5363, 1 µM PI-3K inhibitor BKM120, or DMSO solvent control for 1 hour prior to restimulation with 100 ng/mL OKT3, followed by RICD assessment 24 hours later using propidium iodide staining and flow cytometry. **(C)** Effector T cells were assessed for surface CD3 and **(D)** CD25 expression by flow cytometry on days 4 and 14 post-activation. Statistical tests: One-way repeated measures ANOVA with multiple comparisons (adjusted): (A)control versus AZD5363, p=0.2482; control versus BKM120, p=0.3649. (B) Control versus AZD5363, p=0.0387; control versus BKM120, p=0.0394. For (C) and (D), MFI is plotted for several donors; asterisks denote statistical significance using paired t-tests: (A) p=0.0262. **(**B) p=0.0093.
