# Supplementary figures and images for: TIM-3 drives temporal differences in restimulation-induced cell death sensitivity in effector CD8+ T cells in conjunction with CEACAM1
Source: Cell Death Dis. 2021 Apr 14;12(4):400. doi: 10.1038/s41419-021-03689-6 (PMC8046753; doi:10.1038/s41419-021-03689-6)

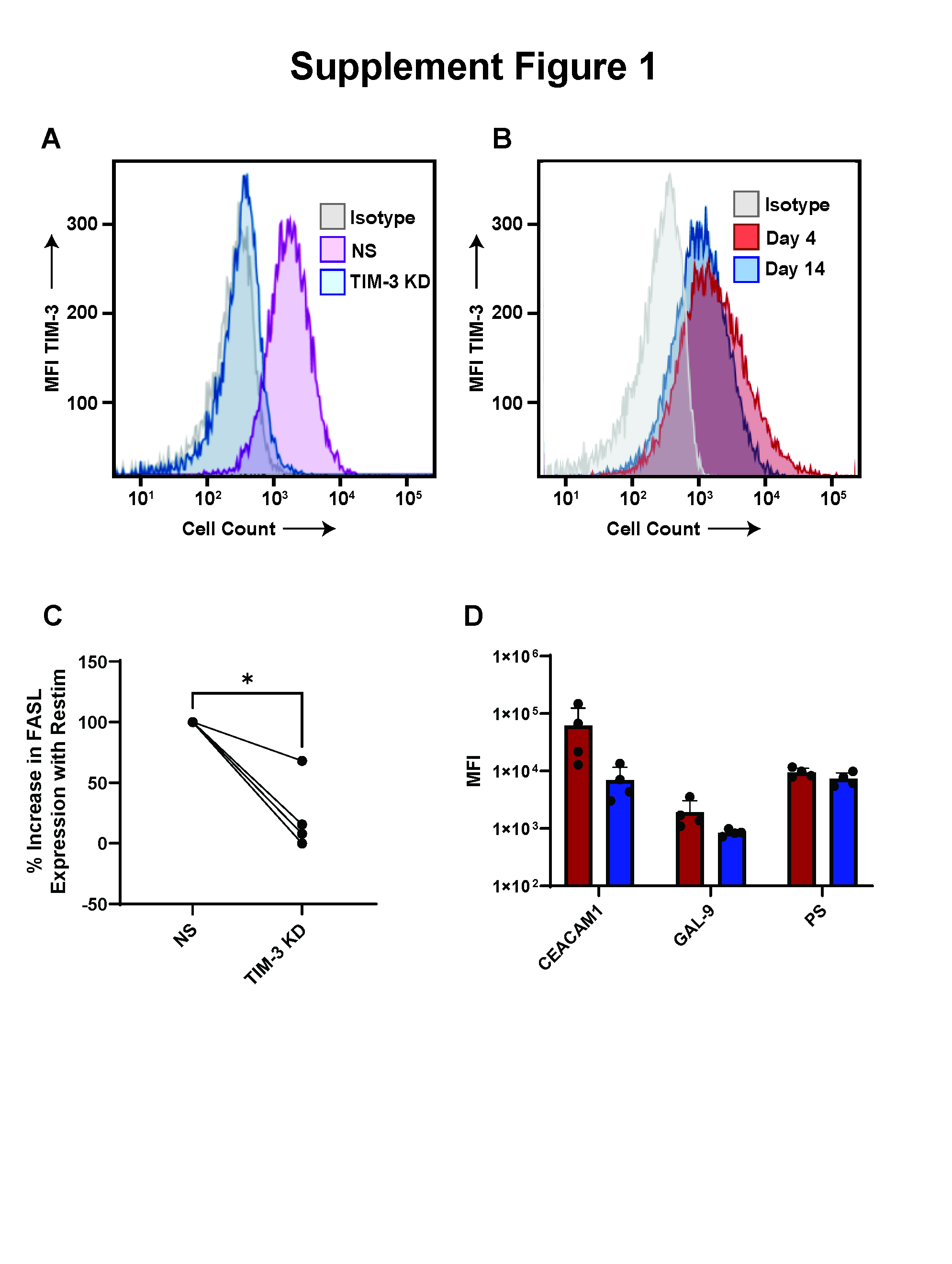

Supplement: Supplementary file 2 — Supplemental Figure 1 [file 41419_2021_3689_MOESM2_ESM.tif]

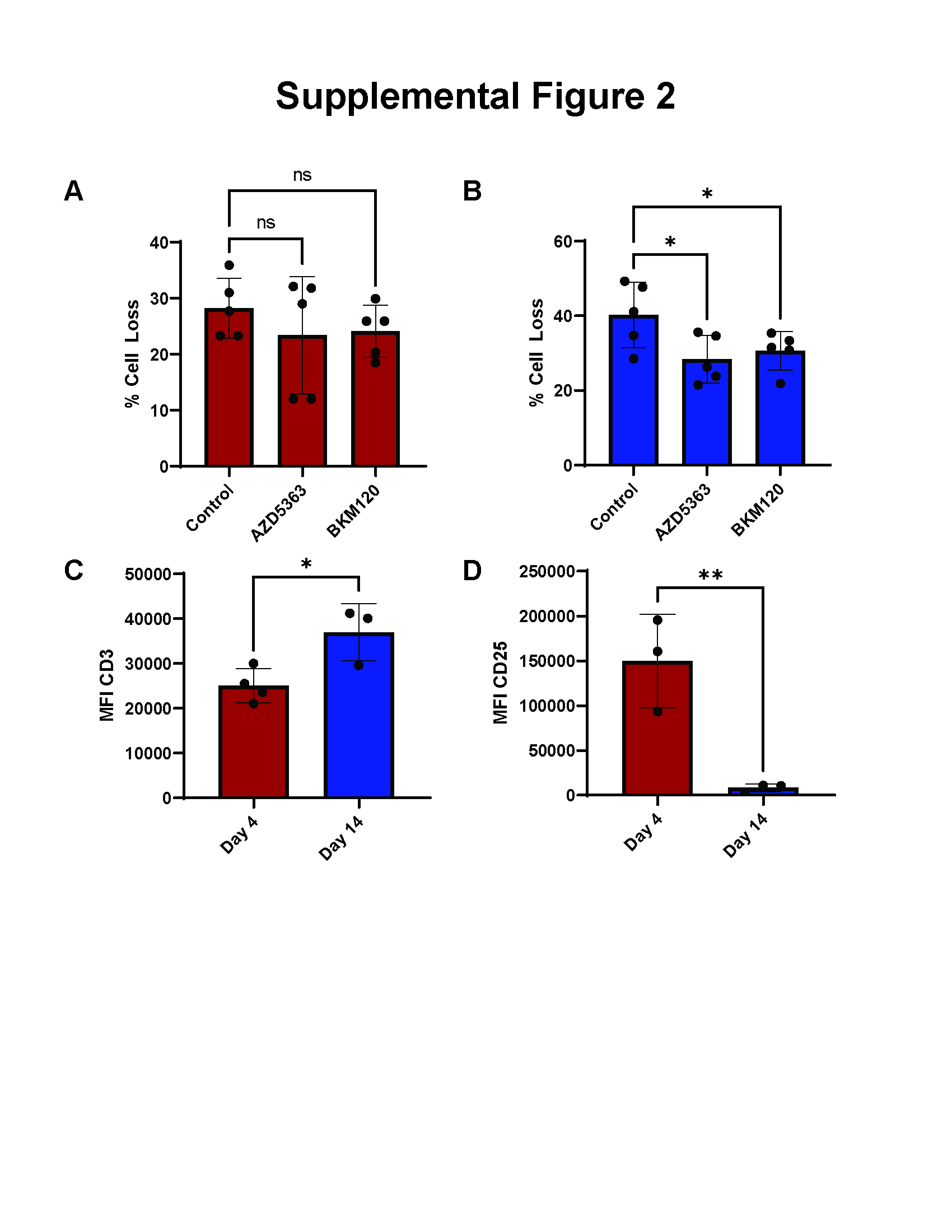

Supplement: Supplementary file 3 — Supplemental Figure 2 [file 41419_2021_3689_MOESM3_ESM.tif]
